# Gene Editing in Pluripotent Stem Cells and Their Derived Organoids

**DOI:** 10.1155/2021/8130828

**Published:** 2021-11-30

**Authors:** Hang Zhou, Yun Wang, Li-Ping Liu, Yu-Mei Li, Yun-Wen Zheng

**Affiliations:** ^1^Institute of Regenerative Medicine, and Affiliated Hospital of Jiangsu University, Jiangsu University, Zhenjiang, Jiangsu 212001, China; ^2^Guangdong Provincial Key Laboratory of Large Animal Models for Biomedicine, and School of Biotechnology and Health Sciences, Wuyi University, Jiangmen, Guangdong 529020, China; ^3^Department of Gastrointestinal and Hepato-Biliary Pancreatic Surgery, Faculty of Medicine, University of Tsukuba, Tsukuba, Ibaraki 305-8575, Japan; ^4^School of Medicine, Yokohama City University, Yokohama, Kanagawa 234-0006, Japan

## Abstract

With the rapid rise in gene-editing technology, pluripotent stem cells (PSCs) and their derived organoids have increasingly broader and practical applications in regenerative medicine. Gene-editing technologies, from large-scale nucleic acid endonucleases to CRISPR, have ignited a global research and development boom with significant implications in regenerative medicine. The development of regenerative medicine technologies, regardless of whether it is PSCs or gene editing, is consistently met with controversy. Are the tools for rewriting the code of life a boon to humanity or a Pandora's box? These technologies raise concerns regarding ethical issues, unexpected mutations, viral infection, etc. These concerns remain even as new treatments emerge. However, the potential negatives cannot obscure the virtues of PSC gene editing, which have, and will continue to, benefit mankind at an unprecedented rate. Here, we briefly introduce current gene-editing technology and its application in PSCs and their derived organoids, while addressing ethical concerns and safety risks and discussing the latest progress in PSC gene editing. Gene editing in PSCs creates visualized *in vitro* models, providing opportunities for examining mechanisms of known and unknown mutations and offering new possibilities for the treatment of cancer, genetic diseases, and other serious or refractory disorders. From model construction to treatment exploration, the important role of PSCs combined with gene editing in basic and clinical medicine studies is illustrated. The applications, characteristics, and existing challenges are summarized in combination with our lab experiences in this field in an effort to help gene-editing technology better serve humans in a regulated manner. Current preclinical and clinical trials have demonstrated initial safety and efficacy of PSC gene editing; however, for better application in clinical settings, additional investigation is warranted.

## 1. Introduction

Pluripotent stem cells (PSCs), such as embryonic stem cells (ESCs) and induced pluripotent stem cells (iPSCs), are extensively used and considered to be viable cellular therapies against complicated and malignant diseases, like leukemia [1]. Additionally, autologous stem cells, when used as a gene therapy vehicle, can minimize host vs. graft responses and facilitate the correction of mutated genes, consequently correcting an enzyme/protein deficiency and treating a variety of diseases [2]. For instance, gene editing in hematopoietic stem cells (HSCs) has been shown to correct the genotype of transfusion-dependent *β*-thalassemia in human cells [3, 4]. Furthermore, transplantation of gene-edited HSCs and progenitor cells (HSPCs) to a leukemia patient with a simultaneous HIV-1 infection was proven to be safe [5]. These outcomes encourage more work in the field of genetic therapy for inherited and currently incurable diseases.

Gene editing is broadly applied in disease modeling [6], exploring disease mechanisms [7] and disease targeting treatments [8]. Jennifer Doudna and Emmanuelle Charpentier, who pioneered gene-editing technology, were awarded the 2020 Nobel Prize in Chemistry, driving an unprecedented boom in the field [9]. Although gene editing is leading to breakthroughs in regenerative medicine and represents a major innovation in medical technology, several challenges remain, including ethical issues and off-target effects. In 2018, the controversial case of a Chinese team who modified the CCR5 gene in the embryonic cells of a pair of twin babies through clustered regularly interspaced short palindromic repeat (CRISPR)/Cas9 gene-editing technology sparked intense debate [10]. The scientists were attempting to provide the babies with partial immunity to HIV; however, the experiment raised serious ethical issues surrounding genetic manipulation, especially given that the genetically edited twins were exposed to potentially detrimental and fatal mutations. This case also serves as a warning that despite the continuous development of gene-editing technology, the challenges of targeted deletion, retention of foreign genetic material, and viral infection can result in unpredictable health hazards. This technology can aid the progression of medicine only when used under strictly controlled parameters.

The applications and potential expansion of gene editing of PSCs and their derived organoids are endless. Here, we systematically analyze and compare several gene-editing methodologies and provide examples of how gene editing has been used in the treatment of diseases, construction of disease models, and exploration of disease mechanisms. Combined with the experiences and ongoing work in our lab, we have expounded the perspectives as well as opportunities associated with gene editing in PSCs and their derived organoids.

## 2. Superiority of PSCs and Gene Editing for Precision Medicine and Therapy

### 2.1. PSCs and Their Organoids

PSCs are self-renewing with infinite proliferation and multipotency. In 2006, Shinya Yamanaka was the first Japanese scholar to use a viral vector to introduce four transcription factors (Oct4, Sox2, Klf4, and c-Myc) into somatic cells to obtain iPSCs, which revolutionized the field of regenerative medicine [11]. Like ESCs, iPSCs are pluripotent and can proliferate indefinitely. However, unlike ESCs, iPSCs are generated from somatic cells and do not have ethical implications; more importantly, they allow for the isolation of patient-derived cells that carry all of the genetic alterations that cause a specific disease. Patient-derived cells provide an experimental system for the construction of patient-derived disease models for pathogenesis investigation and drug screening, as well as cell-based transplantation therapies [12]. Organoids derived from PSCs are three-dimensional cell masses that contain multiple differentiated cells that are highly similar to the respective organ or tissue; thus, they have an advantage in imitating the developmental process of human organs. Such organoids reflect the human environment more comprehensively than conventional stem cells, enabling the identification of pathological mechanisms that more accurately resemble physiological conditions, owing to their consanguinity advantage over animal models. Therefore, PSCs and their derived organoids, which possess irreplaceable advantages over other models, have already contributed much to this field, including in the treatment of heart valve disease [13] and severe acute respiratory syndrome coronavirus 2 (SARS-CoV-2) [14]. Hence, PSCs and their derived organoids have established their position in the field of precision medicine.

### 2.2. Current Status of Gene Editing

Genome editing tools can be divided into four types that are described here according to the timeline of their discovery from the earliest to the most recent: meganucleases (MegNs, also termed homing endonucleases), zinc finger nucleases (ZFNs), transcription activator-like effector nucleases (TALENs), and CRISPR RNA-guided nucleases. The characteristics of each of these four editing tools relative to existing genetic technologies, as well as their advantages and disadvantages, are summarized in Table 1.

Meganucleases (MegNs) rely on the length of the target sequence and the structure of the DNA contact surface to specifically, accurately, and effectively identify the target. The mechanism of DNA recognition by MegNs involves binding patterns of protein side chains and nucleotide bases [15], deformation of groove dimensions, electrostatic distribution of the molecular surface, and additional contacts within and near the minor groove [16]. Binding affinity and cleavage activity sometimes have different efficiencies. Identifying a relatively good performing MegN can consume enormous time and cost [17]. Different substrates change the activity and/or specificity of the inherent function of MegNs [18], illustrating the importance of the context dependent protein–DNA interactions.

Zinc finger nucleases are constructed by fusing a DNA cleavage domain, like the Type II restriction enzyme FokI, to a zinc finger protein (ZFP) [19], enabling it to cleave the target DNA recognized by the ZFP. Four key amino acid residues of the *α*-helix specifically contact each base of the DNA target site; altering these residues allows for the targeting of any desired sequence. However, the intermolecular interaction among individual zinc fingers alters the binding force with the DNA, making the optimization of assembling and testing multiple pairs of ZFN engineering extraordinarily complex.

Transcription activator-like effector nucleases (TALENs) evolved from transcription activator-like (TAL) effectors, which are transcription activators that have peculiar properties of DNA recognition. The monomeric protein chains of TALENs bind DNA in a right-handed spiral manner, without inducing any bend or other substantial structural distortion. Each base is recognized by a highly conserved sequence of typically 33–35 amino acids. Based on the one-to-one corresponding relationship [20], it is relatively easy to assemble a specific identification domain. TALENs exhibit relatively high precision and flexibility.

CRISPR, clustered regularly interspaced short palindromic repeats, is named for the conserved primitive sequence structure of the bacteria and archaea immune defense system [21]. CRISPR-associated protein 9 (Cas9) is an enzyme with cutting and nucleotide-binding protein domains. Cas9 binds to a single guide RNA (sgRNA), which is engineered by fusing CRISPR RNA (crRNA) and transactivating crRNA (tracrRNA) into a single RNA molecule. If the CRISPR RNA (crRNA) is followed by a protospacer adjacent motif (PAM), the complementary target DNA sequence is precisely sheared. In the editing process, the RNA-DNA interaction is the cornerstone of DNA recognition, which differs from MegNs, ZFNs, and TALENs. The superiority of this approach in gene editing is that synthesis of a sgRNA is the only component researchers need to construct; thus, complicated protein domain manipulation is no longer needed.

Among gene-targeting nucleases, MegNs are the most difficult to synthesize. However, they exhibit small sizes, single-chain structures, and high specificity. TALENs are good at targeting specific individual DNA base pairs without affecting the activity or binding force of the nucleases. Only a pair of TALENs can accurately bind to a double-strand break, which may result in a low probability of off-target effects. Engineering and redesigning specific recognition of DNA-binding proteins are a challenging area of research and development. Proteins and DNA have different molecular interface compositions, and their complex relationships include directional hydrogen bonds, electrostatic contacts, ordered solvent molecules, and bound counterions, making protein–DNA interactions elusive and unpredictable. The CRISPR/Cas9 system is the most operable tool because of its RNA-DNA recognition characteristics, which avoids complex protein engineering.

## 3. Applications of Gene Editing in PSCs and Their Organoids

### 3.1. *Ex Vivo* Organoid Models and beyond

The combination of stem cell and gene-editing technologies has led to new innovations in the field of medicine, opening up a new wave of personalized and precision medicine. The creation of organoid disease models through genetic engineering and gene-editing technologies has led to the elucidation of underlying mechanisms of major diseases, with clinically translatable applications.  Table 2 summarizes the more mature research applications of current gene-editing technologies in basic medicine. Both gain- and loss-of-function phenotype disease models can be created by CRISPR/Cas9 in human iPSCs, serving as an efficient tool for human genetic functional studies and drug screening [22].

#### 3.1.1. Visualization of Cell Fate

PSC-derived organoid models can be used to visually trace the fate of cells through development or growth, by constructing knockin reporter genes for specific target genes. To study vocal dysphonia, caused by vocal fold (VF) disorder, a hiPSC-derived VF model with a GFP reporter was transfected via TALEN to simulate the development of VF epithelial cells in utero. This system consisted of a 3D *in vitro* visualized system for VF mucosal disease modeling [23]. To trace the process of melanocyte development and reconstitution into structured tissue, visualizing melanocytic stem cells is key; this is an active area of research in our lab.

#### 3.1.2. Functional Evaluation

To elucidate physiological mechanisms, gene editing and PSC differentiation models may be a perfect combination. Through CRISPR/Cas9-based genome editing technology, key segmentation-clock gene expression showed phase changes in the hPSC-derived presomitic mesoderm. This provided insights into the human segmentation clock related to diseases associated with human axial skeletogenesis [24]. Targeting endogenous genes in hPSCs with small molecule-assisted shut-off helped reveal how FOXG1 syndrome gene dosage affects the generation of neurotransmitter [25]. CRISPR/Cas9 gene-editing produced 11 variants of the HCM-causing mutation in genome-edited human pluripotent stem cell-cardiomyocytes (hPSC-CMs). The main hallmarks of HCM were exhibited through phenotypic rescue and functional evaluation, providing novel putative diagnostic biomarkers and gene-based therapeutic targets for HCM [26].

#### 3.1.3. Role of Pathogenic Genes

The ability to selectively modify genes is important to identify the role of genes in specific pathological changes. In one study, genetically modified hPSCs were generated by CRISPR/Cas9 editing revealing that noncoding gene variants have undeniable effects on *GATA6* gene expression and penetrance during pancreatic agenesis [27]. Using CRISPR/Cas9, the *DISC1* gene in iPSCs was modified, altering the relationship among molecular function, risk factors, and the particular cellular context in psychiatric diseases [28]. Increased cardiac microtissue contraction was caused by CM-associated TNNT2 variants, thus, revealing the gene variants associated with hypertrophic and dilated CMs [29]. Using CRISPR/Cas9, QKI-deficient hESCs (hESCs-QKI [del]) were generated. The analyses of the physiological role of QKI in CM differentiation, maturation, and contractile function demonstrated that QKI was a critical alternative splicing regulator in human cardiogenesis and heart function [30]. Neurooncological ventral antigen 1 (NOVA1) plays a critical role in neural development. The reintroduction of the archaic allele into hiPSCs using CRISPR/Cas9 technology revealed a discrepancy between controls and edited hiPSC-derived cortical organoids. This suggested that NOVA1 may have functional consequences for human neural phenotype evolution [31]. Susceptibility to herpes simplex virus-1 (HSV-1) of hPSC-derived cortical neurons with *SNORA31* mutations is increased, revealing the neuron-intrinsic immunity mechanism of HSV-1 infection [32].

#### 3.1.4. Mechanism Exploration of Known Mutations

To clarify the mechanism of action of known mutations, gene-editing interventions were carried out on pathogenic genes in patient-derived iPSCs or organoids. Mutations in *TSC1* or *TSC2* are known to disturb multisystem development in tuberous sclerosis complex (TSC) [33]. Blair et al. established TSC models using CRISPR-Cas9, and second-hit somatic mutations were found to have an essential effect on the large heterogeneity of tuber number and size among TSC patients [33]. Similarly, the pathogenesis of Cockayne syndrome was revealed using the gene-corrected CS-iPSC (GC-iPSC) model [34]. Knocking out different regions of the TTN gene, mutations in which are known to cause familial dilated cardiomyopathy, revealed that Cronos is crucial to sarcomere formation in human CMs [35]. iPSC-derived cardiac myocytes with KO mutations, mediated by TALENs, could also provide a platform for studying biological function and the pathology of genetic variants in cardiovascular diseases [36].

#### 3.1.5. Functional Exploration of Unknown Mutations

To explore unknown mutations and their effects, comparing PSC models with and without mutations may be effective. For instance, comparing CRISPR/Cas9-based gene editing in hPSC-derived neurons and isogenic controls, it was determined that the internal mechanism of neuronal network dysregulation was due to the *V337M* tau mutation impairing the cytoskeleton in the axon initial segment [37]. Amyotrophic lateral sclerosis (ALS) has long been seen as an energy metabolism-related disease. In another study, using iPSC-derived motor neuron (MN) as a disease model and CRISPR/Cas9 as a tool to correct FUS mutations, surprisingly, metabolic dysfunction was found to not be the underlying cause of the ALS-related phenotypes [38]. Establishing a stepwise model of congenital neutropenia to acute myeloid leukemia (AML), derived from congenital neutropenia patient-derived iPSCs by CRISPR/Cas9, revealed that BAALC and MK2a phosphorylation may be excellent targets for preventing leukemogenic transformation or eliminate AML blasts [39].

#### 3.1.6. Screening for Pathogenic Genes

To screen for unknown genes responsible for diseases, creating mutations and overexpressing or inhibiting gene expression in PSCs and organoid models could help clarify and define key genes of interest. Using CRISPR/Cas9 in hPSCs with an E50K mutation in the optineurin (*OPTN*) gene resulted in differentiation of the cells into retinal ganglion cells. This method establishes an *in vitro* model of neurodegeneration and provides the opportunity to develop novel therapeutic approaches for glaucoma [40]. Generating TREM2 mutation hPSCs using CRISPR/Cas9 in human microglia-like cells, demonstrated TREM2 expression related to amyloid plaque metabolism, which might advance the current understanding regarding Alzheimer's disease (AD) [41]. Moreover, CRISPR/Cas9-mediated FOXO3-enhanced or FOXO3-related protein ablated hESC differentiation into hVSMCs. Vascular protection function was demonstrated for FOXO3, and novel mechanistic insights could be investigated [42]. By knocking out individual 15q13.3 microdeletion genes using CRISPR/Cas9, downstream effects in pathways in neuropsychiatric disorders and interactions between genes were revealed [7]. CRISPR/Cas9 engineered NRL-deficient hESCs- (*NRL* [-/-]) derived retinal organoids demonstrated that NRL is required to define rod identity. Otherwise, S-cone-like cells would develop by default into photoreceptor cell types [43]. *RAP1*-deficient hESCs, also generated with CRISPR/Cas9, revealed that RAP1 may play an important role in aging-associated disorders by telomeric and nontelomeric regulation of cell homeostasis [44].

### 3.2. Mutation Correction and Potential Treatment

Gene editing in PSCs through knockout (KO) or knockin (KI) genes enables observation of phenotypic changes and, potentially, the identification of disease targets for clinical research and therapy.  Table 3 summarizes the uses and existing challenges of gene-editing technologies in the clinical treatment of different diseases.

#### 3.2.1. Immunogenicity Reduction

Immunological rejection is common following organ transplantation. A study based on hiPSC gene editing found that the *ETV2* mutation generates exogenous organs with reduced immunogenicity [82]. In addition, HLA-C-retained immunocompatible donor iPSCs edited by disrupting both HLA-A and -B alleles that evade T cells and natural killer cells *in vitro* and *in vivo* [83].

#### 3.2.2. Patient PSC-Derived Disease Models

Disease models were constructed by PSC differentiation and gene editing. For fragile X syndrome (FXS), an inherited intellectual disability in males, FMR1 was reactivated after the heterochromatin status switched, by targeting demethylation of the CGG expansion using dCas9-Tet1/single guide RNA (sgRNA) in FXS iPSCs. This suggested potential therapeutic strategies for FXS [77]. iPSC-derived cerebral organoids with Alzheimer's disease features and CRISPR/Cas9-edited isogenic lines were used to screen and test blood-brain barrier-permeable drugs; this system may illuminate strategies for precision medicine therapy [8]. Patient iPSC-derived CMs were disputed with *RAF1* mutations by CRISPR/Cas9, and mitogen-activated protein kinase 1/2 (MEK1/2) and extracellular regulated kinase 5 pathways were found to serve as new therapeutic targets to treat HCM [62]. Fibrin-based engineered heart tissue was generated from *DNMT3A* knockout hiPSC-derived CMs. DNA methylation plays an important role in CM development, which suggests that it could be a potential target for cardiac therapy [84]. Generation and subsequent conversion of *CTNS*-KO lines into iPSCs or kidney organoids helped establish disease models. Cystine-depleting drugs were tested in the model [85].

#### 3.2.3. Refractory Disease Models

There are many refractory diseases without effective treatment, some of which are fatal. Although the best therapy cannot be confirmed at once, potential targets can be identified through gene editing carried out on hiPSC models. Both long-QT syndrome and short-QT syndrome are fatal inherited arrhythmogenic syndromes, which can cause apopsychia and death. A human ether-a-go-go-related gene-deficient CM model [6] with a pathogenic mutation, or mutation-corrected hiPSC-CMs [86], was established separately using CRISPR/Cas9, providing clues for malignant hereditary arrhythmia [6]. Moreover, the underlying molecular mechanism of congenital hepatic fibrosis (CHF) remains unclear. PKHD1-KO and heterozygous mutated PKHD1 iPS clones were established. Following analysis of the composition of serum, interleukin-8 (IL-8) and connective tissue growth factor (CTGF) were found to be essential in CHF pathogenesis. Thus, IL-8 and CTGF could be seen as new therapeutic candidate targets for CHF [87]. Based on a CRISPR/Cas9 KO strategy, a study found that adhesion, metastasis, and propagation of somatic cancer cells were closely related to OCT4A, indicating that targeting OCT4A may be a promising combination therapy for human cancers [88]. A significant decrease or increase in the expression of knockin and knockout *PARK2*, respectively, in iPSCs by CRISPR/Cas9 technology revealed that the *PARK2* mutation, related to catechol-O-methyltransferase (COMT), may make a difference in the initial process of Parkinson's disease; treatment with central COMT inhibitors may thus be useful [89]. In another study, researchers combined iPSCs and CRISPR/Cas9 technologies to develop a clonal evolution model of AML. Cell-autonomous dysregulation of inflammatory signaling was identified as an early and persistent event in leukemogenesis, which suggested a promising early therapeutic target [90].

#### 3.2.4. Rescue Models of Definite Etiology

Sometimes, as the basis of a definitive etiology, gene-editing treatment methods can be manipulated in PSC-derived models to identify rescue treatments. PSC-derived alveolar epithelial type 2 cells (AEC2s) provide a platform for disease modeling, exhibit self-renewal capacity, and display additional AEC2 functional capacities. In iAEC2s generated from a child with severe lung disease carrying an *SFTPB* mutation, the mutation was corrected by CRISPR-based gene editing rescued surfactant processing in AEC2s [91]. Calcium ion plays a central role in heart failure development; CRISPR/Cas9-mediated CRISPLD1-KO led to dysregulated Ca^2+^ handling in hPSC-CM. This study provided new evidence on the critical role of Ca^2+^ in heart failure pathophysiology; simultaneously, novel candidate genes were found for therapeutic interventions [92]. Excision of the FXN intron by CRISPR/Cas9 in iPSC-derived dorsal root ganglia organoids rescued molecular and cellular deficits of the disease. This system revealed several pathological mechanisms for repairing complex neuronal circuits [93]. iPSCs carrying a heterozygous K219T mutation in *LMNA* generated an iPSC-based model of LMNA-cardiomyopathy (CMP). When corrected by CRISPR/Cas9, the functional and molecular defects of the disease model were rescued, describing a new pathogenic mechanism for the conduction defects associated with LMNA-CMP [94]. Another study using a similar strategy identified the underlying mechanism of LMNA-CMP conduction abnormalities [95]. Using patient-derived iPSCs and CRISPR/Cas9 engineering to develop a Leigh syndrome (LS) model, mechanistic insights and potential interventional strategies were indicated for a rare mitochondrial disease [96]. Marfan syndrome (MFS) is a genetically inherited connective tissue disorder; a vascular model derived from MFS patient-iPSCs was used to assess the molecular mechanisms. A *FBN1* mutation, a critical pathogenic factor of MFS, was corrected by CRISPR-based editing, and abnormalities of the model were subsequently rescued, thus identifying novel targets for treatment [55]. Splicing defects in cystic fibrosis were corrected by allele-specific genome editing with AsCas12a-crRNA nuclease system, paving the way for a permanent splicing correction in genetic diseases [97].

#### 3.2.5. In Vivo Transplantation to Validation

Some mutations can be rescued by gene editing, and gene-rescued PSCs can differentiate into mature cells and be transplanted into animal models, improving and possibly curing the animal. TWIK-related spinal cord K^+^ channel (TRESK) is implicated in nociception and pain disorders; a CRISPR/Cas9-corrected TRESK function-related mutation, F139WfsX2, showed a reversal in neuronal excitability. This suggests TRESK activators may be a promising therapeutic approach to pain and migraine [98]. iPSC-based cell therapy was developed for Canavan disease by introducing the aspartoacylase (*ASPA*) gene into patient iPSC-derived neural progenitor cells or oligodendrocyte progenitor cells using TALEN-mediated genetic engineering [79]. The approach established in this study provides a robust proof of principle for cell therapy strategies. *BEST1* mutant iPSC-derived retinal pigment epithelium models in the study showed that gene augmentation or gene editing had equal efficacy, which guides some genotypically diverse disorders [99]. In the late stage of diabetes, patients must regularly inject exogenous insulin. In this study, researchers used CRISPR/Cas9 to correct a diabetes-causing pathogenic variant in iPSCs derived from a patient with Wolfram syndrome. After transplantation, the diabetes phenotype was rescued in mice [100]. The iPSC-derived Duchenne muscular dystrophy disruption model using CRISPR/Cas9 offers new options for restoring muscle function, potentially treating patients in the future [101].

### 3.3. Risk Control for Ethics and Off-Target Effects

Ethical issues have always been unavoidable in the context of gene editing [102]. CRISPR/Cas9-mediated adenine base editors can correct STAT3 p.R382W in patient-derived iPSCs, providing a potential treatment for STAT3-hyper IgE syndrome; however, for clinical translation, safety and ethical implications still need to be resolved [103]. For human medical development, ethics should be a priority and appliance stringently monitored, but also not be a stumbling block. The International Society for Stem Cell Research recognized this and permitted heritable changes to the human genome under the premise of safety [104]. Safety must always be a crucial prerequisite for clinical applications. Indeed, various promising stem cell treatments were stopped owing to the carcinogenic potential of the cells. Meanwhile, research using genome-engineering strategies has demonstrated the protective effect of a suicide system for inactivating dividing cells. In this study, human ESCs with homozygous modifications of *CDK1* exhibited normal morphology, self-renewing capacity, and differentiation capacity compared with control hESCs. Researchers also established a system to assess and quantify the safety of cell-based therapies [105]. Although a second-generation PCSK9-specific MegN showed reduced off-target cleavage, it still appeared at ~30 off-target cleavage sites. Cells derived from human iPSCs may provide a perfect *in vitro* model for observing the propensity to cleave at off-target sites [61]. Safeguard mechanisms ameliorate the potential cell therapy risks; for example, one metabolic engineering study using genome editing methods to disrupt uridine monophosphate synthetase generated a transgene-free safety switch for cell therapy [106].

### 3.4. Future Perspectives

Although viral vectors are known to have high delivery efficiency, they can be double-edged swords, with continuous expression of CRISPR/Cas9 nuclease and gRNA causing off-target mutagenesis and immunogenicity. Off-target risk has always been a major concern for genetic treatment; however, through the use of PSC culture and differentiation technology, cells that are deemed to be safe can potentially be used for clinical applications. At the same time, more studies that are committed to safe and efficient gene-editing strategies are needed, similar to those described below.

#### 3.4.1. Transfection

A nanovesicle-based delivery system, NanoMEDIC, delivers large molecules, such as ribonucleoprotein; the nanovesicles are cleared within 3 days [107]. CRISPR/Cas9 RNA-guided endonucleases (RNP) can be transported to certain cells by modifying the surface affinity of the extracellular vesicles for certain cells [108], both of which potentially reduce off-target risks and improve targeting efficiency. Moreover, a technically simple system has been described that employs electroporation to significantly enhance genome targeting capabilities in primary human hematopoietic cells [109].

#### 3.4.2. Base Editing

Cytosine and adenine base editors (CBEs and ABEs) are powerful tools for single-base modification. However, editor components, DNA repair proteins, and local sequence context interact, resulting in unpredictable editing outcomes. Researchers who focused on illuminating base editing have provided refined and novel insights, which may improve the precision of base editing [110]. By applying SpCas9-ABE (PAM recognition sequence: NGG) and xCas9-ABE (PAM recognition sequence: NGN) to cystic fibrosis intestinal organoids, genetic and functional repair was obtained. Furthermore, no off-target mutations were detected, indicating that ABE may be safely applied in human cells [111].

#### 3.4.3. Homology Repair

Nonhomologous end-joining (NHEJ), microhomology-mediated end-joining (MMEJ), and homology-directed repair (HDR) are the three main types of cellular DNA repair machinery. To determine the most efficient HDR strategy, researchers introduced different forms of donor DNA and observed that editing with a 400 bp dsDNA repair template increased the efficiency of repair [112]. Combining the small molecular compounds M3814 and trichostatin A inhibited NHEJ repairs predominantly and increased HDR efficiency, which potentially improves the efficiency of knockins [113]. MMEJ-based therapeutic strategies could be used in diseases that are associated with microduplications [114].

#### 3.4.4. Newly Developed Editing Tools

Prime editing is a genome editing technology combining Cas9-nickase and reverse transcriptase with greater precision than Cas9-mediated HDR. When performed, nearly no off-target effects are observed; thus, it has potential in future clinical applications to safely repair human monogenic diseases [115]. Furthermore, the *Natronobacterium gregoryi*-derived Ago protein demonstrated nickase activity at 37°C [116] five years after the technology had been thrown out. It will be interesting to observe the applications of this technology going forward.

Although various gene-editing methods have emerged, their broad and direct use in clinical settings remains a long road ahead.

## 4. Conclusions

The rapid advancement of genome editing technologies, from MegNs to CRISPR, has improved the operability, efficiency, and safety of gene editing. The combination of gene editing and stem cell technologies has advanced the research and development of the life and medical sciences. Through knockin and knockout technologies, human genetic and pathogenic mechanisms of disease can be better explored, and gene expression and disease progression can be traced. Drug development can also be accelerated, contributing to the advancement of personalized precision gene therapy for inherited diseases.

The existing gene-editing technologies each have their particular characteristics and advantages, but all have some corresponding challenges. Although MegNs have high specificity and low cytotoxicity, they are difficult to manipulate, limited in variety, and time-consuming, and it is expensive to design sequence-specific enzymes. ZFNs, although a relatively mature platform and more efficient than homologous recombination, are highly off-target and cytotoxic, have low specificity, are sequence-dependent upstream and downstream, and are only suitable for *in vitro* manipulation. Although TALENs are easier to design than ZFNs and their targets are not restricted, their modules are cumbersome to assemble, require extensive sequencing work, and are costly and cytotoxic. CRISPR has a high rate of gene modification and diverse gene regulation, enables simultaneous knockdown of multiple targets, is precise in its targeting, has a low off-target rate, is inexpensive, and is easy to operate. However, it still suffers from the inability to cut the pretarget region without PAM, off-target effects, and transfection difficulties.

Although current preclinical trials have demonstrated initial safety and efficacy of gene editing, existing studies have also shown that the immunogenicity and cytotoxicity of these vectors are of concern. Improving the accuracy of detecting and then reducing off-target effects remain a challenge. Only when these problems are solved can gene-editing technology be better applied in the clinical setting.

## Figures and Tables

**Table 1:** Characteristics of current gene editing technologies and their advantages and limitation.

	Identifying patterns	Cleavage domain	Recognition length	Identification conditions	Minimum identification unit	Accuracy	Molecular weight size of editing tools	Operational difficulty	Off-target level	Cytotoxicity	Advantages	Limitations
MegNs	Binds specific DNA through protein-DNA interactions	4 bp	Double-stranded DNA sequences of 12 to 40 base pairs	Monomer, target DNA	Indeterminate	+++	200-400 aa	+++	+	+	Higher specificity	Limited variety; difficult to retrofit
ZFN	Binds specific DNA through protein-DNA interactions	5-7 bp	9-18 bp per ZFN	Dimers, 3 bp units of target DNA	3 bp	++	500-1300 aa	++	++	++	Mature platform; more efficient than homologous recombination	High off-target rate; low specificity; design dependent on upstream and downstream sequences; only for in vitro operations
TALEN	Binds specific DNA through protein-DNA interactions	5-7 bp	14-20 bp per TALEN	Dimer, transcription activator-like effector or transcription activating effector nuclease 5′ preceded by a central structural domain of T	1 bp	++	900-1100 aa	++	++	+++	Unrestricted target sites; easier design than ZFN; higher specificity	Cumbersome module assembly; requires large sequencing effort; high cost
CRISPR	Binding of specific DNA through base complementary pairing and protein-DNA interactions	0 bp	20 bp	Monomer, 3′ sequence for NGG's guide RNA	1 bp	++	1300-1500 aa	+	+	+	High rate of gene modification; diverse gene regulation; allows simultaneous knockout of multiple target loci; precise targeting inexpensive	No PAM in the pretarget region cannot be cut; transfection difficulties

PAM: protospacer adjacent motif.

**Table 2:** Landmarks and trends of gene editing in life and medical sciences.

	Editing methods	Target cells	Targeted genes	Virus transfection	Animal models	Points	Year
MegN	KI	mESCs	Villin locus	Yes	/	Induction of gene-targeting and homologous recombination events	1998 [45]
	M	293T	RAG1 locus	No	/	Targeting endogenous genes; low targeting efficiency; with cytotoxicity	2009 [46]
	KI	293T	*π*10 locus	Yes	/	Delivering meganucleases into cells in a transient and dose-controlled manner; low targeting efficiency; with cytotoxicity	2011 [47]
ZFN	KI/GFP	hESCs	OCT4 locus、AAVS1 locus	Yes	m	Gene targeting in hESCs	2009 [48]
	M	hESCs	Genomic *α*-synuclein locus (SNCA)	No	/	Genome editing in hESCs; off-target detection needs to be improved; targeting efficiency needs to be enhanced	2011 [49]
	KO	hiPSCs	LRRK2 (sigma)	No	m	Parkinson's pathogenesis; patient-derived iPSCs; low targeting efficiency; with cytotoxicity	2013 [50]
	M	hiPSCs	MAPT	Yes	/	Designed mutation iPSCs; FTD pathogenesis; targeting efficiency needs to be enhanced	2018 [51]
	KO	FRT cells	CFTR	No	r	Disease targets; designed KO model	2020 [52]
TALEN	KI/GFP	hPSCs	OCT4 locus	No	/	Genetic engineering for hPSCs; targeting efficiency like ZFN	2011 [53]
	KO	hiPSCs	TNNT2, LMNA/C, TBX5, MYH7, ANKRD1NKX2.5	Yes	/	Human-based KO cell model *in vitro*; greater freedom and flexibility in target site selection than CRISPR	2017 [36]
	KI/GFP	hiPSCs	AAVS1 locus	No	/	3D organoid models; GFP; mechanistic studies	2019 [23]
CRISPR	KO	hiPSCs	AAVS1 safe harbor locus	No	/	TetO inducible system; feasibility and reversibility of CRISPRi; high off-target efficiency	2016 [54]
	M	hiPSC	FBN1	No	/	Vascular models, human iPSCs; pathogenesis of MFS	2017 [55]
	KI/GFP	hESCs	gRNAs made from the lentiGuide-puro construct	Yes	/	A genome-scale screening; hESCs; impaired differentiation	2019 [56]
	KO	hPSCs	NRL	No	/	A 3D organoid model; disease pathogenesis; high targeting efficiency	2021 [43]

ESCs: embryonic stem cells; FTD: frontotemporal dementia; FRT: Fischer rat thyroid; h: human; iPSCs: induced pluripotent stem cells; KI: knockin; KO: knockout; m: mouse; M: mutation; MFS: Marfan syndrome; PSCs: ESCs and iPSCs; r: rat.

**Table 3:** Current challenges of gene editing in different diseases.

	Disease names	Related genes	Editing technologies	Model types	Clinical trials	Challenge points and limitation	Years
Respiratory disorders	CF	CFTR	CRISPR	Organoids	\	Proof of concept only, gene editing off-target effects; needs further evaluation for safety	2013 [57]
			TALEN	Cells	\	Delivery efficiency needs to be improved; targeting accuracy needs to be improved	2019 [58]
			CRISPR	Patient-derived cells	\	Difficulty of in vivo delivery, genetically corrected airway stem cell transplantation and recovery of in vivo mucus cilia transport	2021 [59]
	NSCLC	PD-1	CRISPR	\	Phase I (first)	Underexpansion and low response rate of T cells after gene editing; small study sample	2020 [60]
Circulatory disorders	HC	Protein PCSK9	MegNs	Macaques	\	Off-target effects, with cytotoxicity, immunogenicity to be overcome	2018 [61]
	NS-associated HCM	RAF1	CRISPR	Patient-derived cells	\	RAF1 lacks a nuclear localization sequence (NLS), its translocation mechanism is unknown, and the molecular mechanism of the disease needs to be further explored	2019 [62]
	HC	Ldlr	CRISPR	Mouse	\	Genome editing efficiency to be improved and off-target effects to be overcome	2020 [63]
	NS-associated HCM	LZTR1	CRISPR	Patient-derived cells	\	Proof of concept only, needs in vivo evaluation, patient-specific iPSC-CM model is still immature and needs to be improved	2020 [64]
	LDS	TGFBR1	CRISPR	Patient-derived cells	\	Needs further proof from in vivo experiments, off-target effects	2021 [65]
Infectious diseases	HIV	CCR5	ZFN	\	Yes	A serious adverse event was associated with the infusion of ZFN-modified autologous CD4 T cells, with off-target safety issues to be overcome	2014 [66]
			ZFN	Mouse	\	Reduced proliferation of editorial cells transplanted in vivo, delivery efficiency and targeting accuracy need to be improved	2013 [67]
			TALEN	Cells	\	Delivery efficiency and targeting accuracy need to be improved	2015 [68]
			CRISPR	Mouse	\	Safety issues to be further assessed	2017 [69]
			CRISPR	\	Yes	Off-target efficiency needs to be improved, targeting accuracy needs to be improved, and generalizability needs to be further assessed	2019 [5]
			CRISPR	Patient-derived cells	\	Off-target efficiency needs to be improved, and targeting accuracy needs to be improved	2020 [70]
Hematologic disorders	TDT & SCD	BCL11A	CRISPR	\	Yes	No comprehensive genomic analysis of clinical samples and the generalizability of the results needs to be further determined	2021 [71]
	TDT & SCD	HPFH5	CRISPR	Cells	\	Off-target effects to be overcome and safety to be improved	2016 [72]
	SCD	HBB	CRISPR	Mouse	\	The off-target efficiency needs to be reduced, and more sensitive off-target analysis methods are needed	2019 [73]
	SCD	HBB	CRISPR	Humanized mouse	\	Delivery methods to be optimized and delivery efficiency to be improved	2021 [74]
	ALL	CD52	TALEN	\	Yes	Immunogenicity needs to be further reduced; safety needs to be further tested; small sample size	2017 [75]
	MM	TRAC、CD52	TALEN	Mouse	\	Delivery efficiency needs to be improved, and long-term safety issues need to be further studied	2019 [76]
Neurological disorders	FXS	FMR1	CRISPR	Mouse	\	The off-target efficiency needs to be reduced, more sensitive off-target analysis methods are needed, and safety issues need to be further tested	2018 [77]
	AD	TREM2	CRISPR	Humanized SCD mouse	\	Further analysis of the mechanism of action is needed to find effective therapeutic targets for disease treatment	2020 [78]
	CD	ASPA	TALEN	Mouse	\	Proof of concept only, how to achieve sustained efficacy remains to be addressed, and the issue of safety still needs to be improved	2020 [79]
Ophthalmology	XLRP	RP2	CRISPR	Organoids	\	Retinal-like organs are still immature and need further improvement	2020 [80]
	LCA10	CEP290	CRISPR	Mouse	Yes	Impact of individual differences on safety of off-target effect delivery, durability of efficacy to be further assessed	2019 [81]

AD: Alzheimer's disease; ALL: acute lymphocytic leukemia; CD: Canavan disease; CF: cystic fibrosis; FXS: fragile X syndrome; HC: hypercholesterolemia; HCM: hypertrophic cardiomyopathy; iPSC-CMs: iPSC-derived cardiomyocytes; LDS: Loeys-Dietz syndrome; LCA10: Leber congenital amaurosis type 10; MM: multiple myeloma; NS: Noonan syndrome; NSCLC: non-small-cell lung cancer; SCD: sickle cell disease; TDT: transfusion-dependent *β*-thalassemia; XLRP: X-linked retinitis pigmentosa.
